# *Atarbolana makranensis*, a new species of Cirolanidae (Crustacea, Isopoda) from Makran, Iranian coast of the Gulf of Oman

**DOI:** 10.3897/zookeys.522.6056

**Published:** 2015-09-23

**Authors:** Valiallah Khalaji-Pirbalouty, Reza Naderloo, Alireza Keikhosravi

**Affiliations:** 1 Department of Biology, Faculty of Science, Shahrekord University, Shahrekord, Iran; 2 School of Biology, College of Science, University of Tehran, Tehran, Iran; 3 Department of Biology, Institute of Animal Science, University of Hakim Sabzevari, Iran

**Keywords:** Isopoda, Cirolanidae, *Atarbolana*, new species, Gulf of Oman, Iran

## Abstract

*Atarbolana makranensis* **sp. n.** is described from the intertidal zone of Makran along the Iranian coast of the Gulf of Oman. *Atarbolana makranensis* **sp. n.** can be recognized by the presence of a tuft of long setae on the antennal flagellum of males, elongate pleotelson with 12 robust marginal setae, pleotelson with narrowly rounded apex extending well beyond the uropodal endopod, uropodal endopod half as long as exopod with 14 robust marginal setae, and appendix masculina with an acute apex and extending beyond endopod distal margin. A key is provided for the four known species of *Atarbolana* Bruce & Javed, 1987.

## Introduction

The isopod fauna of the Gulf of Oman is poorly studied; in particular, there is no record of this group from the northern coast of the Gulf of Oman. Among the different families, the family Cirolanidae has received little attention in the southern coastlines of Iran. Recently, Khalaji-Pirbalouty and Wägele (2011) described two cirolanid isopods, *Baharilana kiabii* and *Cirolana tarahomii*, from the Qeshm and Kish islands along Iranian coast of the Persian Gulf.

**Figure 1.**
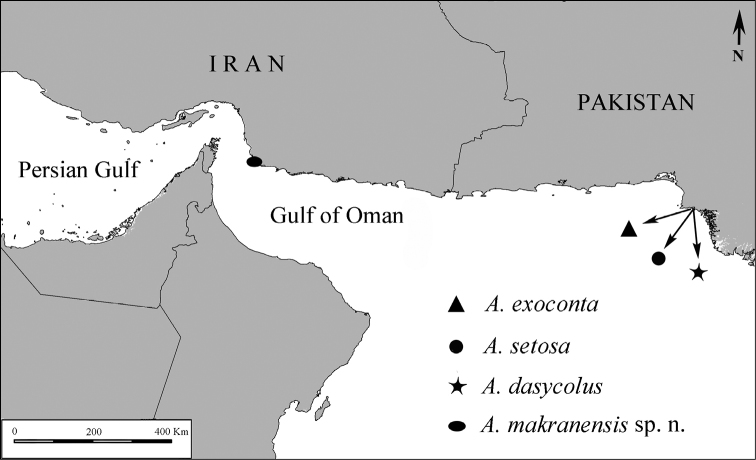
Geographical distribution of *Atarbolana* species.

The genus *Atarbolana* Bruce & Javed, 1987, was established with the description of *Atarbolana exoconta* from the rocky intertidal coast of Manora Island, Pakistan. *Atarbolana setosa* Javed & Yasmeen, 1989, and *Atarbolana dasycolus* Yasmeen, 2004, have been subsequently described from the same coast (Karachi, Pakistan). *Atarbolana makranensis* sp. n. constitutes the fourth species of the genus from the northwestern Indian Ocean and is the first species of the order Isopoda recorded from the Iranian coast of the Gulf of Oman (Fig. 1).

## Materials and methods

Specimens for this study were collected by turning over rocks and washing algae and sea grasses. The material was preserved in 96% ethanol and has been deposited in the Zoological Museum Hamburg, Hamburg, Germany (ZMH), and the Zoological Museum, University of Tehran, Iran (ZUTC). Appendages were dissected and fixed in stained antibacterial glycerine-gelatine (Merck). Drawings were made with the aid of a camera lucida attached to Olympus BX 51 and were then processed using Corel Draw (version X5) and Adobe Photoshop (version CS5). Terminology of the morphological characters follows that of Khalaji-Pirbalouty and Bruce (2014).

### Abbreviations

AM Australian Museum;

ZMH Zoologisches Museum Hamburg, Hamburg, Germany;

ZUTC Zoological Museum, University of Tehran, Iran;

PMS plumose marginal setae;

RS robust seta/setae;

CPS circumplumose setae,

SPS sensory palmate seta/setae.

## Taxonomy

### Suborder Cymothoida Wägele, 1989 Family Cirolanidae Dana, 1852

#### Genus Atarbolana

Taxon classificationAnimaliaIsopodaCirolanidae

Bruce & Javed, 1987

Atarbolana Bruce & Javed, 1987: 145; Javed and Yasmeen 1989: 78; Yasmeen 2004: 21.

##### Type species.

*Atarbolana exoconta* Bruce & Javed, 1987; by designation and monotypy.

##### Type locality.

Manora Island, Karachi, Pakistan.

##### Species included.

*Atarbolana setosa* Javed and Yasmeen (1989), and *Atarbolana dasycolus* Yasmeen, 2004.

##### Diagnosis.

Diagnoses to the genus are to be found in Bruce and Javed (1987) and Javed and Yasmeen (1989).

##### Remarks.

In addition to the generic diagnosis given by the above authors, pereopod 7 has a flattened merus and carpus with numerous long plumose setae. Female is similar to male but smaller on average. As stated by Bruce and Javed (1987), females differ from males (apart from primary sexual characteristics) by having a shorter and less setose antennal flagellum. Pleotelson elongation is less than that in males and in most cases number of robust setae are less than in those of males. In contrast to the diagnosis given by Bruce and Javed (1987), uropod rami of females are smaller than that of males; they are subequal and extending almost to the level of pleotelson apex or slightly extending beyond. Brood pouch composed of five pairs of oostegites arising on sternites 1–5.

There are several characters that exclude the species of this genus from *Cirolana* Leach, 1818, and other cirolanid genera. These characters are a cylindrical uropod exopod, an oval uropod endopod, the uropod peduncle with a row of robust setae along the ventral margin, and in having a reduced pleon.

The genera *Eurylana* Jansen, 1981, and *Pseudolana* Bruce, 1979, with appendix masculina inserted medially, and short penes, appear to be most similar to *Atarbolana*. However, *Eurylana* has no secondary unguis on the dactylus and is readily separated from congers by the morphology of the clypeal region and pleopods. *Pseudolana* differs in having a linear frontal lamina, five visible pleonites and a wide pleotelson (Bruce 1986). In addition, *Atarbolana* has endopods of pleopods 3–5 entirely without marginal setae. Only few genera like *Anopsilana* Paulian & Deboutteville, 1956 have such character, but with appendix masculine arising basally and absence of penes.

#### Atarbolana makranensis sp. n.

Taxon classificationAnimaliaIsopodaCirolanidae

http://zoobank.org/C4C1D285-83E9-4EAE-94D6-8BB6F3703856

**Figure 2.**
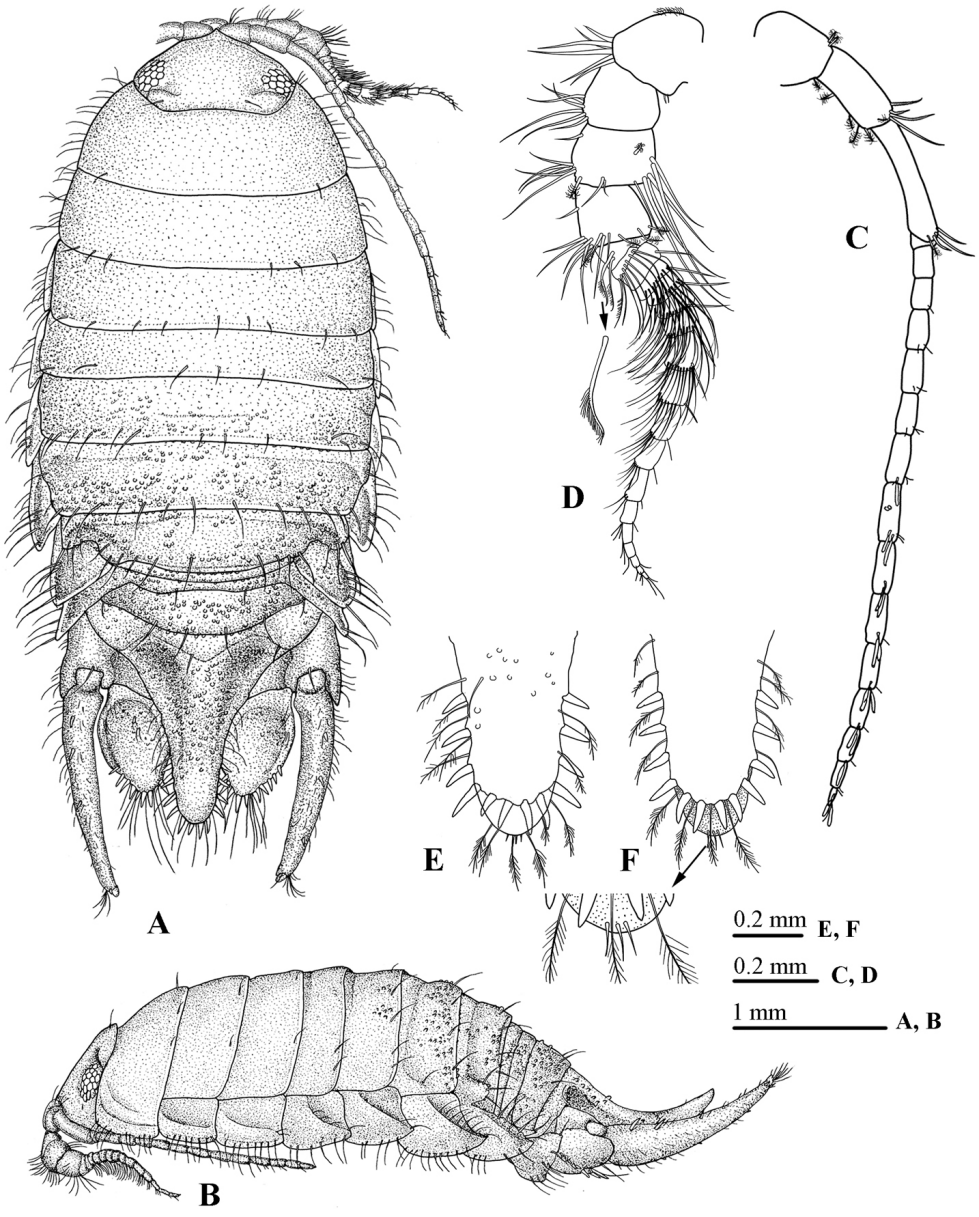
*Atarbolana makranensis* sp. n., male, holotype (ZMH–K–42597). **A** dorsal view **B** lateral view **C** antennule **D** antenna **E** pleotelson apex (dorsal view) **F** pleotelson apex (ventral view).

**Figure 3.**
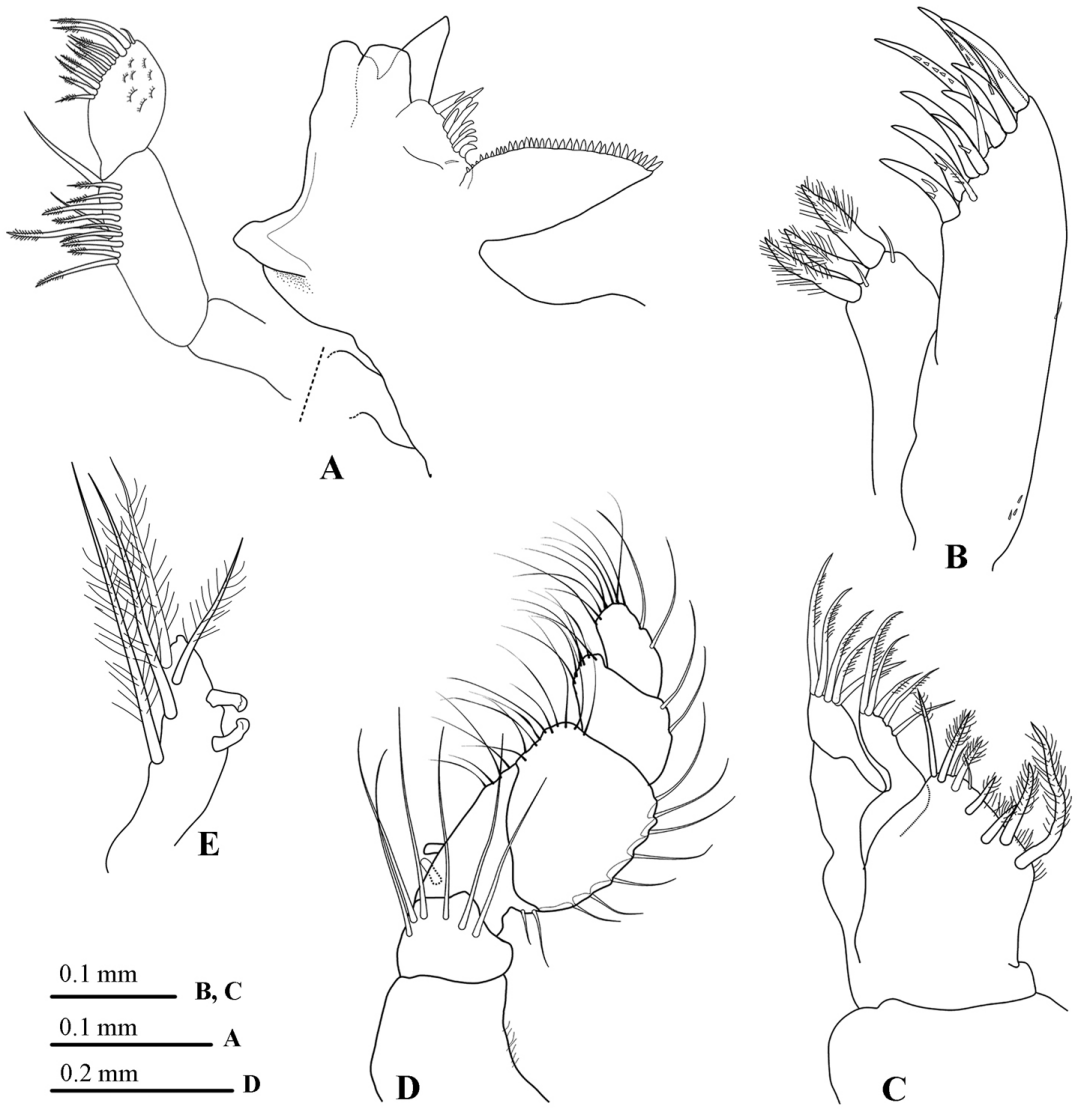
*Atarbolana makranensis* sp. n., male, holotype (ZMH–K–42597). **A** left mandible **B** maxillule **C** maxilla **D** maxilliped **E** maxilliped endite.

**Figure 4.**
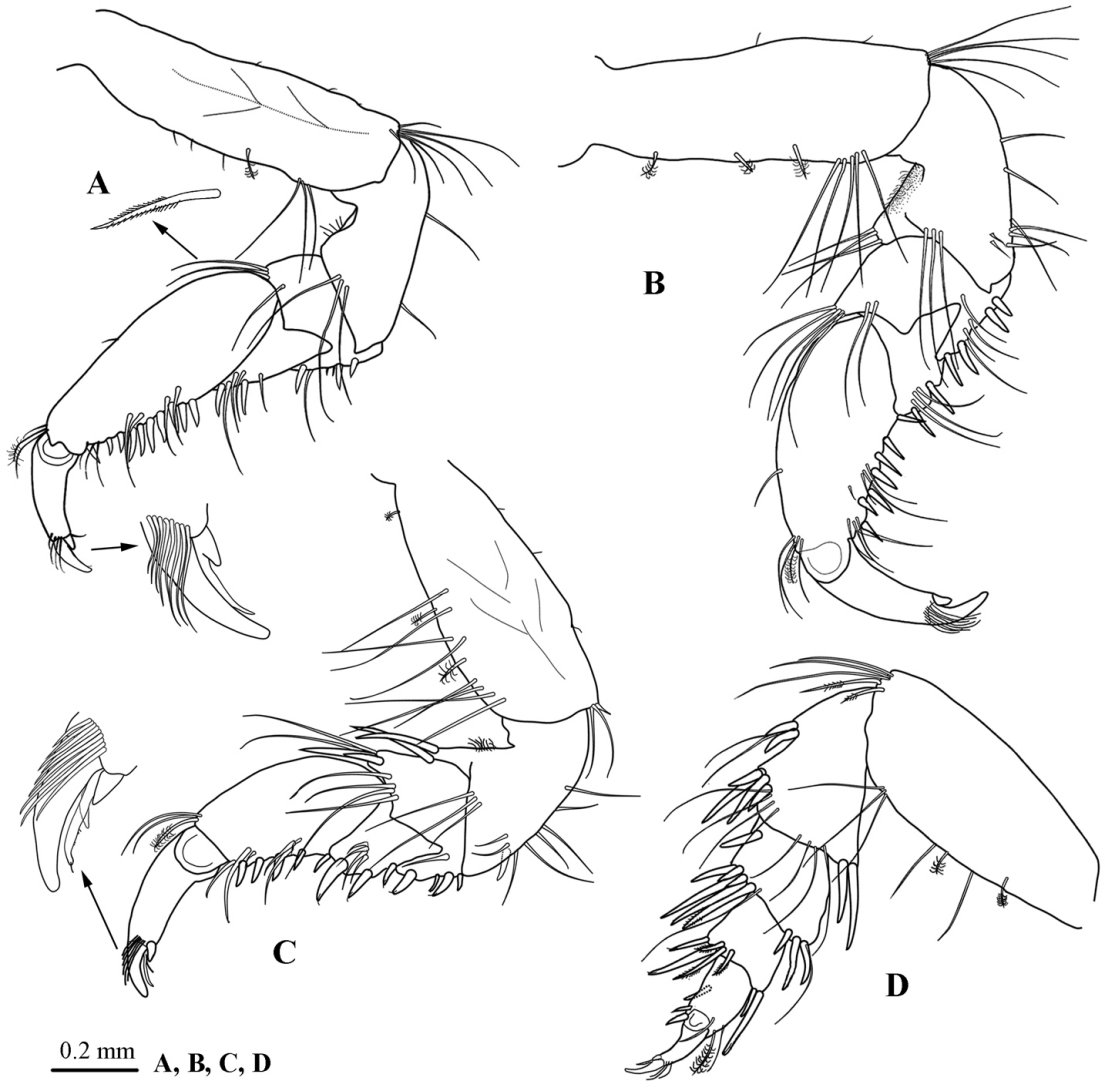
*Atarbolana makranensis* sp. n., male, holotype (ZMH–K–42597). **A–D** pereopods 1–4 respectively.

**Figure 5.**
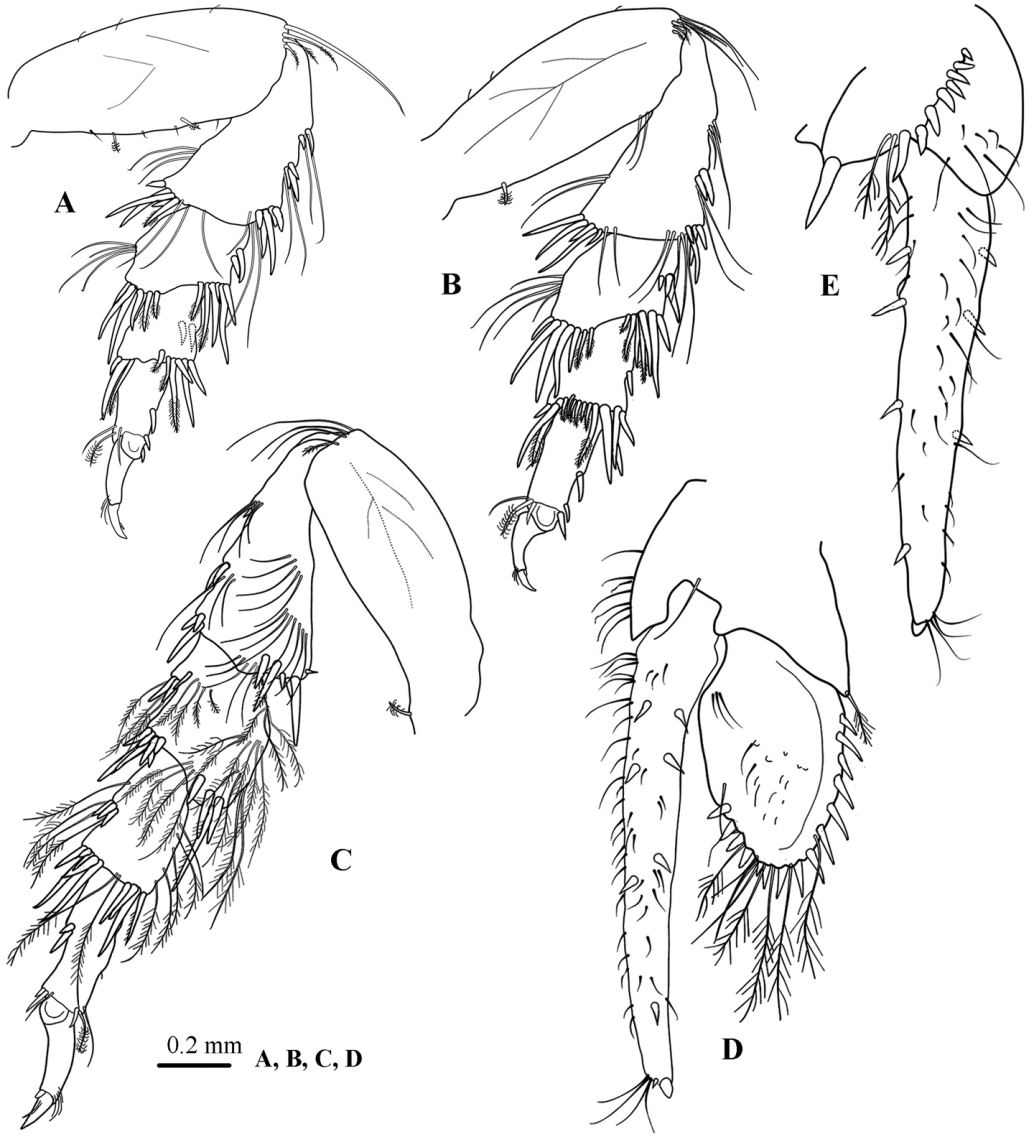
*Atarbolana makranensis* sp. n., male, holotype (ZMH–K–42597). **A–C** pereopods 5–7 respectively **D** uropod dorsal view **E** uropod ventral view.

**Figure 6.**
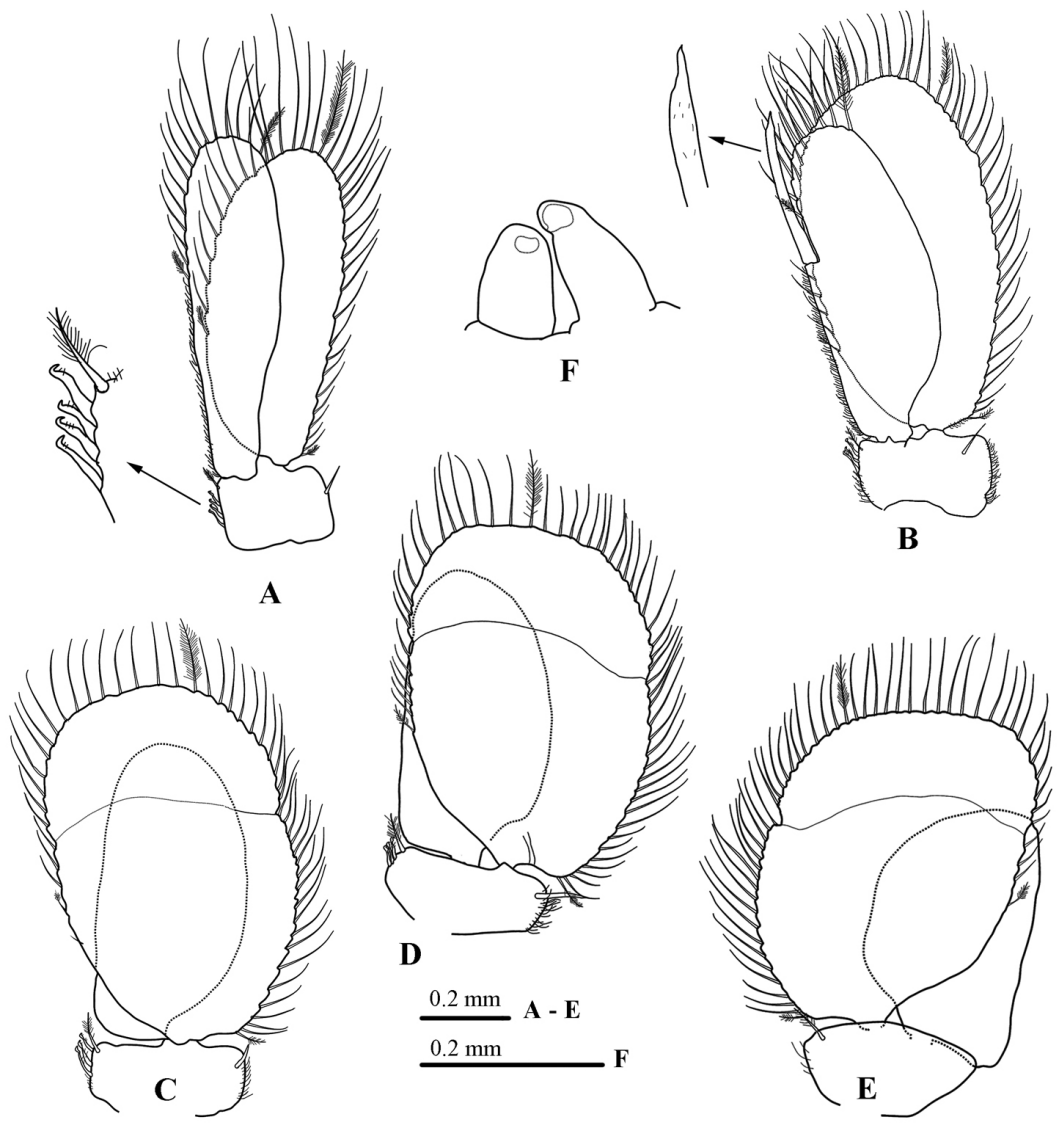
*Atarbolana makranensis* sp. n., male, holotype (ZMH–K–42597). **A–E** pleopods 1–5 **F** penes.

Figs 2 , 3 , 4 , 5 , 6

##### Material examined.

*Holotype*: ♂ (4.4 mm) Gatan-Paein, Hormuzgan Province, Iran, Gulf of Oman, rocky intertidal shore covered with algae, 25°58'1.52"N, 57°15'13.78"E, 27 December 2013, coll. V. Khalaji-Pirbalouty, R. Naderloo (ZMH–K–42597). *Paratypes*: 5 ♂♂ (4.9, 4.4, 3.9, 3.8, 3.7 mm), 1♀ (ovig. 3.5mm), 9 ♀♀ (3,0–3.8 mm), same data as holotype (ZUTC 5481); 1♂ (3.6 mm), 4 ♀♀ (2.5, 2.6, 2.8, 3.0 mm), same locality as holotype, 30 June 2013, coll. V. Khalaji-Pirbalouty, R. Naderloo (ZUTC 5482).

##### Diagnosis.

Body 2.3 times as long as greatest width; pereonites 5–7, pleon and pleotelson bearing scattered small tubercles; flagellar articles 1–10 in male bearing a tuft of long serrate and simple setae; pleotelson elongated, with narrowly rounded apex, posterior margin with 12 marginal RS; uropod peduncle ventro-mesial surface with a row of 8 RS, uropodal endopod not reaching to pleotelson apex, with 14–15 (left/right) marginal RS, lateral margin proximally lacking RS, exopod about two times as long as endopod; appendix masculina with an acute apex, arising above mid-point of endopod medial margin, and extending slightly beyond endopod distal margin.

##### Description of male.

Body 2.3 times as long as greatest width, widest at pereonite 5 (Fig. 2A). Head with acute rostral point, with 2 sutures posteriorly. All pereonites posterior margins bearing long simple marginal setae; pereonite 1 with 2 curved furrows laterally (Fig. 2B); pereonites 5–7 bearing scattered small tubercles; pereonites 2–3 with sub-quadrate coxal plates; coxal plates 5–7 progressively more produced and acute posteriorly, produced beyond posterior margin of respective segment; all coxal plates with entire, oblique carina, all coxal plate ventral margin fringed with long simple setae (Fig. 2B).

*Pleon* (Fig. 2A) with pleonite 1–2 concealed by pereonite 7; pleonite 3 visible dorsally; pleonite 4 extended well over lateral margins of pleonite 5 and proximal part of uropodal peduncles, pleonites 3–5 with scattered small tubercles.

*Pleotelson* (Fig. 2A, E, F) progressively upturned into a narrowly rounded apex, with 2 bimedian depressions, dorsal surface with scattered small tubercles; posterior margin bearing 12 marginal RS set between long PMS; apical marginal RS concealed by apical margin, distally with 3 small marginal setae (Fig. 2E, F).

*Antennule* (Fig. 2C) extending to posterior margin of pereonite 3, peduncle article 1 short, peduncle article 3 approximately 1.5 times as long as article 2; flagellum with 15 articles, articles 7–15 bearing 1 or 2 aesthetascs.

*Antenna* (Fig. 2D) peduncle articles 2–4 subequal in length, all articles each with a group of long simple setae on antero-distal corner, article 3 with 5–6 very long simple setae on ventral margin; flagellum with 17 articles, extending to posterior margin of pereonite 1, articles 1–10 bearing a tuft of long setae (some serrated), distoventral corner with a single long simple seta.

*Left mandible* (Fig. 3A) molar process anterior margin with about 33 flat teeth; spine row composed of 9 spines; palp article 2 longest with 10 robust biserrate setae and 2 robust simple setae, article 3 with 10 robust biserrate marginal setae.

*Maxillule* (Fig. 2B) lateral endite with 10 RS (weakly serrated) and 2 slender setae; mesial endite with 3 large circumplumose RS and 2 short simple setae.

*Maxilla* (Fig. 3C) lateral and middle endites each with 5 long finely plumose setae; mesial endite with 7 long circumplumose RS and 2 small simple RS.

*Maxilliped palp* (Fig. 3D) article 1 with 6 distally placed long setae, articles 2–5 lateral margins with 2, 7, 2 and 2 slender simple setae respectively; articles 3– 5 with continuous fringe of finely biserrate setae on medial margin; endite (Fig. 4E) with 4 long CPS, and 2 coupling hooks.

*Pereopod 1* (Fig. 4A) *basis* 3.4 times as long as wide, superior margin with 3 long simple and 2 SPS, posterodistal angle with 3–5 long finely plumose setae; *ischium* inferior margin with 2 long simple setae, mediodistal margin with 3 long simple setae; *merus* inferior margin with 3 RS and 1 long simple setae, medio-distal margin with 1 long simple setae, superior margin with 3 long simple setae; *carpus* triangular, inferior margin with 2 RS and 4 long simple setae; *propodus* inferior margin with 10 RS and 3 sets of sub-marginal slender simple seta, superior margin with a single simple seta, superodistal angle with 2 simple and 1 plumose setae; *dactylus* with minute secondary unguis, bearing a transverse row of 8 simple setae at base.

*Pereopod 2* (Fig. 4B) *basis* 2.5 times as long as wide, superior margin with 5 long simple and 3 SPS, posterodistal angle with 5 long finely plumose or simple setae; *ischium* inferior margin with 6 long simple setae, mediodistal margin with 3 long simple setae, superior margin with 3 long simple setae; *merus* inferior margin with 4 RS and 4 long simple setae, superodistal angle with 5 long setae, mediodistal margin with 2 long simple setae; *carpus* triangular, inferior margin with 2 RS and 3 long simple setae; *propodus* inferior margin with 7 robust and 2 sets of sub-marginal slender simple setae, superior margin with a single simple seta, supero-distal angle with a SPS and 3 simple setae; *dactylus* with minute secondary unguis, bearing a transverse row of several simple setae at base.

*Pereopod 3* (Fig. 4C) similar to *pereopod 2*.

*Pereopod 4* (Fig. 4D) *basis* 2.3 times as long as wide, with 5 long simple submarginal and 2 SPS, postero-distal angle with 3 long simple setae and 2 long finely biserrate setae; *ischium* supero-distal angle with 2 long RS, supero-medial surface with 4 long simple setae, inferior margin with 2 sets of RS and 2 sets of long simple sub-marginal setae; *merus* inferior margin with 2 sets of RS (1 + 7), superior distal angle with 3 RS and 1 long simple seta; *carpus* inferior distal margin with 6 simple or serrated RS, superior distal angle with 2 RS; *propodus* inferior margin with 3 RS, superior distal angle with 1 SPS and 2 simple setae; *dactylus* with minute secondary unguis, sub-marginal row of 3 simple setae.

*Pereopod 5* (Fig. 5A) and *Pereopod* 6 (Fig. 5B) are similar to pereopod 4 as illustrated.

*Pereopod 7* (Fig. 5C) *basis* 2.5 times as long as wide, postero-distal angle with 1 serrated RS and 4 long simple setae; *ischium* superior distal angle with 5 biserrate or simple RS, medial surface with 3 rows of long simple setae, inferior margin with 3 sets RS and 2 sets long simple sub-marginal setae; *merus* and *carpus* superior and inferior margins fringed with numerous long plumose setae; *propodus* inferior margin with 2 sets robust setae, superior distal angle with 1 RS, I simple and 1 SPS; *dactylus* with minute secondary unguis.

*Pleopod 1* (Fig. 6A) exopod and endopod with ~39 and 16 PMS, endopod longer and narrower than exopod; sympod 1.5 times as wide as long, mesial margin with 4 coupling hooks and 1 plumose seta, lateral margin with a single RS.

*Pleopod 2* (Fig. 6B) exopod and endopod with ~54 and 10 PMS respectively; *appendix masculina* arising above 0.6 of endopod medial margin, extending slightly beyond endopod distal margin (by approximately 0.2 times of length), tapering to an acute apex; sympod mesial margin with 3 coupling hooks and 1 plumose seta, lateral margin with a single sub-marginal RS.

*Pleopod* 3 (Fig. 6C) and *Pleopod* 4 (Fig. 6D) essentially similar, exopod with ~51 and ~58 PMS, and an entire transverse suture; sympod medial margin with 3 coupling hooks and 1 plumose seta, lateral margin with single sub-marginal RS.

*Pleopod* 5 (Fig. 6E) exopod with 56 plumose marginal setae, and entire transverse suture; sympod without coupling hook, lateral margin with single sub-marginal RS.

*Penes* (Fig. 6F) short, separate but adjacent, 2.5 times as long as basal width.

*Uropod* (Fig. 5D); *endopod* not reaching to pleotelsonic apex, with 14–15 (left/right) marginal RS, lateral margin proximally lacking RS; *exopod* (Fig. 5E) elongate, slender, nearly 2 times as long as endopod, extended well beyond pleotelsonic apex, dorso-lateral margin with 3 RS, ventro-medial margin with 4 RS, distal margin with a tuft of long simple setae, 1 prominent RS and 1 small RS; peduncle ventral side with a single long RS distally, ventro-medial surface with a row of 8 RS and 2 long plumose setae.

*Female.* Apart from sexual characters differs from male by having an antenna without tuft of long setae on flagellum articles; uropod rami smaller than in male and extending just beyond the pleotelsonic apex, endopod with 12 marginal RS (rather than 14 in male); pleotelson with 8 marginal RS (rather than 12 in male), elongation less than in male.

##### Variations.

Pleotelson marginal RS (n = 14 [7♂ and 7♀]) males with 10–12 RS, with 12 RS (86%) most frequent, and 10 (14%) occurring only once; in females with 8–9 RS, with 8 RS most frequent (71%), and 9 (28%). Uropod endopod in males with 14–15 RS, with 14 RS (86%) most frequent, and 15 (14%) occurring only once; in females with 12–13 RS, with 12 RS (86%) most frequent, and 13 (14%) occurring only once.

##### Remarks.

*Atarbolana makranensis* sp. n. can be identified by the elongate pleotelson with a narrow apex and 12 marginal RS in male. The ventral surface of the uropod peduncle with a row of 8 RS, uropodal endopod not extending to apex of the pleotelson, about half length of exopod, lateral margin lacking RS proximally. *Atarbolana makranensis* sp. n. is similar to *Atarbolana setosa* Javed and Yasmeen (1989), and *Atarbolana dasycolus* Yasmeen, 2004 (both described from Karachi, Pakistan), in having an antennal flagellum with tufts of long and dense setae. However, the two species can be clearly distinguished from *Atarbolana makranensis* by having an elongated appendix masculina which extends well beyond the apex of the endopod of the pleopod 2. Furthermore, in the new species the pleotelson extends well beyond the uropodal endopod, whereas in *Atarbolana setosa* the pleotelson extends just to the endopod apex. *Atarbolana dasycolus* has a pleotelson with 8 marginal RS and does not extend to the endopod apex. In addition, in *Atarbolana makranensis* the uropod exopod/endopod ratio is approximately 2, whereas it is 1.7 in *Atarbolana setosa* and 1.35 in *Atarbolana dasycolus*. Based on the drawings and description of *Atarbolana exoconta*, the type species of the genus, given by Bruce and Javed (1987) and examination of paratype material (AM. P.37200, P.37276, Manora Island, Pakistan), *Atarbolana exoconta* differs from *Atarbolana makranensis* in having a shorter pleotelson with 16 marginal RS, lacking long setae on the antennal flagellum, and a lower uropod exopod/endopod ratio (1.34).

##### Etymology.

The specific epithet of the new species refers to its type locality, Makran, which is the name of the area with the original Aryan people living in the southeast of Iran along the coast of the Gulf of Oman.

### Key to the species of *Atarbolana*

**Table:** 

1	Appendix masculina extending well beyond pleopod 2 endopod distal margin (by ≥ 0.5 its length)	**2**
–	Appendix masculina extending slightly beyond pleopod 2 endopod distal margin (by ≤ 0.2 its length)	**3**
2	Pleotelson with 10 marginal RS, elongated, extending just beyond the uropodal endopod distal margin	***Atarbolana setosa***
–	Pleotelson with 8 marginal RS, short, not extending to uropodal endopod distal margin	***Atarbolana dasycolus***
3	Antennal flagellum of adult male lacking long setae, pleotelson short, with 16 marginal RS	***Atarbolana exoconta***
–	Antennal flagellum of adult male with tufts of long and dense setae, pleotelson elongated, with 12 marginal RS	***Atarbolana makranensis* sp. n.**

## Supplementary Material

XML Treatment for Atarbolana

XML Treatment for Atarbolana makranensis
